# A circuit‐based approach to modulate hypersexuality in Parkinson's disease

**DOI:** 10.1111/pcn.13523

**Published:** 2023-02-03

**Authors:** David Mata‐Marín, José Ángel Pineda‐Pardo, Mario Michiels, Cristina Pagge, Claudia Ammann, Raúl Martínez‐Fernández, José Antonio Molina, Lydia Vela‐Desojo, Fernando Alonso‐Frech, Ignacio Obeso

**Affiliations:** ^1^ Centro Integral de Neurociencias Abarca Campal (HM CINAC) Hospital Universitario HM Puerta del Sur. HM Hospitales Madrid Spain; ^2^ Network Center for Biomedical Research on Neurodegenerative Diseases (CIBERNED) Instituto Carlos III Madrid Spain; ^3^ PhD program in Neuroscience Autonoma University of Madrid Madrid Spain; ^4^ Hospital 12 de Octubre Madrid Spain; ^5^ Hospital Fundación Alcorcón Madrid Spain; ^6^ Hospital Clínico San Carlos Madrid Spain; ^7^ Department of Psychobiology & Methods for the Behavioral Sciences Department Complutense University of Madrid Madrid Spain

**Keywords:** dopamine, impulse‐control disorders, neuroimaging, Parkinson's disease, transcranial magnetic stimulation

## Abstract

**Aim:**

Impulse‐control disorder is a common neuropsychiatric complication in Parkinson's disease (PD) under dopamine replacement therapy. Prior studies tested the balance between enhanced desire towards reward and cognitive control deficits, hypothesized to be biased towards the former in impulse control disorders. We provide evidence for this hypothesis by measuring behavioral and neural patterns behind the influence of sexual desire over response inhibition and tools towards functional restoration using repetitive transcranial stimulation in patients with hypersexuality as predominant impulsive disorder.

**Methods:**

The effect of sexual cues on inhibition was measured with a novel erotic stop‐signal task under on and off dopaminergic medication. Task‐related functional and anatomical connectivity models were estimated in 16 hypersexual and 17 nonhypersexual patients with PD as well as in 17 healthy controls. Additionally, excitatory neuromodulation using intermittent theta‐burst stimulation (sham‐controlled) was applied over the pre‐supplementary motor area in 20 additional hypersexual patients with PD aiming to improve response inhibition.

**Results:**

Compared with their nonhypersexual peers, patients with hypersexuality recruited caudate, pre‐supplementary motor area, ventral tegmental area, and anterior cingulate cortex while on medication. Reduced connectivity was found between pre‐supplementary motor area and caudate nucleus in hypersexual compared with nonhypersexual patients (while medicated), a result paralleled by compensatory enhanced anatomical connectivity. Furthermore, stimulation over the pre‐supplementary motor area improved response inhibition in hypersexual patients with PD when exposed to sexual cues.

**Conclusion:**

This study, therefore, has identified a specific fronto‐striatal and mesolimbic circuitry underlying uncontrolled sexual responses in medicated patients with PD where cortical neuromodulation halts its expression.

Impulse control disorder (ICD) is a distressing neuropsychiatric complication in patients with Parkinson's disease (PD) receiving dopaminergic replacement therapy. The most prevalent forms include compulsive shopping, pathological gambling, hypersexuality, and excessive eating, all of which are marked by a tendency to compulsively engage in excessive and inappropriate actions towards rewarding items. ICDs are reported to have a prevalence of ~17% to 60% in patients with PD induced by dopamine agonists^1^, ^2^ and also, more rarely, by levodopa.^3^ Critically, ICD may become severe enough to put patients at increased risk of financial ruin, marital and family disruption, prosecution, and job‐related problems. To date, treatment follows two clinical routes: reducing dopaminergic doses at the expense of worsening parkinsonism or deep brain stimulation to reduce dopaminergic medication while maintaining movement capacity.^4^, ^5^ Hence, no convenient medical treatment is currently available for ICD in PD.

ICD in PD is mainly understood as the result of dopaminergic dysregulation of the mesolimbic pathway, including the orbital prefrontal cortex, anterior cingulate cortex (ACC), ventral striatum (nucleus accumbens), amygdala, and ventral tegmental area (VTA) mediating reward.^2^ Mesolimbic dysregulation is thought to result from dopaminergic denervation of the ventral striatum, as shown by decreased dopamine transporter availability in patients with ICD compared with patients with non‐ICD PD correlating with ICD severity.^6^, ^7^ Indeed, a recent model has suggested that dopaminergic reduction of the ventral striatum is a relevant predisposing factor to develop ICD in patients with PD.^8^ Accordingly, ICD may be understood as a state of excessive approach secondary to enhanced desire to reward items that find little or no opposition.^9^ The dysregulated limbic system in PD and ICD may produce such excessive desire toward rewards, possibly by interfering with cognitive control operations.

To date, it is unclear whether changes in response inhibition, enhanced desire, or their interaction underlie ICD. We recruited patients with PD with hypersexuality (PD + HS) and designed a novel paradigm combining erotic and nonerotic cues within a stop‐signal task to investigate the behavioral, neural, and connectivity (anatomical and functional) profile in PD + HS (study 1). Hypersexuality is one of the most common subtypes of ICD (13.6%^3^), thus we included hypersexual patients and used sexual images that elicit high physiological responses adaptable to magnetic resonance imaging (MRI) contexts.^10^ Hence, since reactive response inhibition is vulnerable to high‐arousal stimuli,^11^, ^12^ we hypothesized that sexual cues will specifically bias reactive response inhibition driven by changes along fronto‐striatal networks in patients with PD + HS. To test this, we employed two imaging techniques: dynamic causal modeling (DCM) to assess changes in effective connectivity, and diffusion tensor imaging (DTI) to examine potential underlying changes in brain anatomy. Next, we hypothesized that response inhibition in patients with PD + HS can be improved by means of repetitive transcranial magnetic stimulation (rTMS, study 2) over the pre‐supplementary motor area (pre‐SMA), a critical hub for exerting inhibitory control of behavior.^13^, ^14^ The circuit‐based approach hereby utilized may serve to elucidate the neurobiological changes when response inhibition is influenced by sexual desire in a specific subtype of ICD and offer novel therapies to a yet untreatable disturbance.

## Materials and Methods

### Participants

Our sample included 16 PD + HS (age: 62.50 ± 9.1 years), 17 PD without hypersexuality (PD – HS; age: 65.40 ± 5.4 years), and 17 healthy controls (age: 58.12 ± 9.8 years) in study 1 and 20 PD + HS in study 2 (age: 57.89 ± 8.0 years). Patients in both studies were heterosexual men and right‐handed with no difference in terms of disease duration or medication intake and similar age and educational level among them or when compared with healthy controls (final sample details in Table 1). Patients met UK Brain Bank criteria for diagnosis of PD. Patients were recruited at HM‐CINAC clinics and other three movement disorders units from Madrid (Spain). Enrollment was confirmed after an initial interview with the neuropsychologist (with both patients and families) to confirm heterosexuality and hypersexuality (≥5 on the sexual subscale of the Questionnaire for Impulsive‐Compulsive Disorder in Parkinson's Disease [QUIP]). The PD – HS group was selected based on absent or limited presence of ICD‐related behaviors while on medication (<4 on the sexual subscale of the QUIP). Since hypersexuality is one of the most frequent subtypes of ICD, mostly in men,^15^ we designed the task using female erotic stimuli, and we limited the recruitment to heterosexual male participants. Exclusion criteria were established based on presence of: (i) cognitive dysfunction (≤24 on the Montreal Cognitive Assessment); (ii) depression (>18 in Beck Depression Inventory); or (iii) other severe comorbidities (e.g. history of substance abuse, hallucinations, or psychosis). The study was approved by the hospital's ethics committee (17.03.0852E2‐GHM) and written informed consent was obtained from all participants prior to study onset.

**Table 1:** Demographic, clinical, and neuropsychological data for each group

	Study 1				Study 2
	PD + HS (*n* = 14)	PD − HS (*n* = 14)	Controls (*n* = 16)	Stats	PD + HS (*n* = 18)
Age (years)	62.50 ± 9.1	65.40 ± 5.4	58.12 ± 9.8	0.96	57.89 ± 8.0
Education (years)	12.50 ± 4.3	12.93 ± 3.9	14.35 ± 3.4	0.69	12.71 ± 2.9
Disease duration (years)	8.38 ± 3.2	6.27 ± 3.9	‐	0.14	6.89 ± 4.5
UPDRS‐III (off)	29.31 ± 7.4	27.80 ± 10.4	‐	0.41	28.65 ± 11.4
UPDRS‐III (on)	15.13 ± 5.1	14.53 ± 6.2	‐	0.73	17.47 ± 7.71
LEDD (total)	740.63 ± 273.3	549.29 ± 293.2	‐	0.12	738.61 ± 631.2
LEDD (DA)	206.67 ± 84.3	171.82 ± 76.4	‐	0.44	362.67 ± 74.3
LEDD (L‐dopa)	583.33 ± 237.3	527.27 ± 262.0	‐	0.52	375.94 ± 286.8
QUIP total	16.24 ± 11.9	5.27 ± 8.5	7.88 ± 10.6	<0.01	30.89 ± 14.99
Hypersexual	6.41 ± 3.9	1.20 ± 1.7	1.47 ± 1.8	<0.001	8.61 ± 3.78
Gambling	0.65 ± 1.2	0.20 ± 0.5	0.29 ± 0.9	0.21	1.00 ± 2.17
Shopping	1.76 ± 2.3	0.67 ± 1.4	1.47 ± 1.7	0.12	3.83 ± 4.45
Binge eating	2.76 ± 3.3	0.67 ± 1.1	1.46 ± 1.9	0.02	4.33 ± 2.99
Hobbyism	1.35 ± 2.2	1.00 ± 1.9	1.65 ± 2.3	0.63	4.56 ± 4.02
Pounding	1.24 ± 2.3	0.67 ± 1.8	1.47 ± 2.3	0.45	4.11 ± 3.72
Medication	2.12 ± 3.6	0.87 ± 1.7	0.06 ± 0.2	0.23	4.44 ± 4.08
MoCa	27.67 ± 1.5	28.00 ± 1.3	27.42 ± 1.2	0.41	27.94 ± 2.1
Stroop	79.6 ± 37.3	62.6 ± 14.5	51.18 ± 16.5	0.10	57.89 ± 8.1
FAB	17.33 ± 0.9	16.93 ± 1.8	17.94 ± 0.2	0.24	16.88 ± 1.8
GDS	7.92 ± 4.4	8.77 ± 6.1	3.59 ± 2.4	0.59	8.18 ± 7.1
BAI	11.85 ± 5.0	14.69 ± 8.5	6.94 ± 4.3	0.29	12.06 ± 10.0
SAS	10.08 ± 4.7	12.15 ± 8.8	4.29 ± 4.6	0.46	11.94 ± 6.4
BIS‐III	79.50 ± 18.5	73.69 ± 12.6	81.08 ± 12.8	0.39	58.44 ± 8.4
BSFI	26.82 ± 10.2	21.00 ± 7.1	23.75 ± 12.5	0.11	12.71 ± 2.9

*Note*: Stats: unpaired *t*‐test between Parkinson disease with hypersexuality (PD + HS) and Parkinson disease with hypersexuality (PD – HS), between groups.

Abbreviations: BAI, Beck's Anxiety Inventory; BIS‐III, Barratt Impulsiveness Scale; BSFI, Brief Sexual Function Inventory; DA, dopamine agonist (total mg); FAB, Frontal Assessment Battery; GDS, Geriatric Depression Scale; L‐dopa, levodopa (mg); LEDD, levodopa equivalent daily dose (total mg); MoCa, Montreal Cognitive Assessment; QUIP, Questionnaire for Impulsive‐Compulsive Disorder in Parkinson's Disease; SAS, Starkstein Apathy Scale; UPDRS‐III, Unified Parkinson's Disease Rating Scale.

### Procedure

In study 1, patients were assessed in the on‐ and off‐medication states inside the functional MRI (fMRI) scanner with at least 1 week between sessions and no more than 10 days apart (counterbalanced order). The off‐state was defined as overnight dopaminergic drug withdrawal of 12 h (range: 10–14 h). Additionally, scores of arousal (Data S1) were collected to measure sexual desire (Table 2).

**Table 2:** Sexual desire pre‐task vs post‐task performance and exposure to sexual visual stimuli

	Pre‐task	Post‐task	*P‐*values
**Off session**			
PD + HS	2.76 ± 1.1	4.43 ± 1.3	<.001^a^
PD − HS	0.90 ± 0.6	1.10 ± 0.7	.18^a^
*P*‐values	<.001^b^	<.001^b^	
**On session**			
PD + HS	2.74 ± 0.9	5.65 ± 1.0	<.001^a^
PD − HS	0.60 ± 0.5	0.90 ± 0.5	.09^a^
*P*‐values	<.001^b^	<.001^b^	

*Note*: Results obtained from average values of the first two items on the arousal scale.

Abbreviations: PD + HS, Parkinson disease with hypersexuality; PD – HS, Parkinson disease without hypersexuality.

^a^ Paired *t*‐test comparison.

^b^ Unpaired *t*‐test comparison.

A single‐blind randomized controlled study was designed to assess the acute effects of rTMS (study 2) over the pre‐SMA (target selected from group‐level results of study 1). Each patient underwent real and sham stimulation sessions in separate days at least 1 week apart and no more than 10 days apart (counterbalanced order) during on medication. Immediately after stimulation, patients performed the erotic stop‐signal task described below. Behavioral measures (classic stop‐signal task), neuropsychological, and neuropsychiatric tests were performed during on medication.

### The erotic stop‐signal task

We designed an erotic stop‐signal task using sexual cues (two parallel versions; using an existing database^15^ and additional images). Erotic and nonerotic images were rated based on valence and arousal states in a pre‐experiment pilot phase by healthy heterosexual male participants (*n* = 22; age: 48.4 ± 2.5 years). We then selected the most consistent high‐valued images to present during the task.

**Fig. 1:**
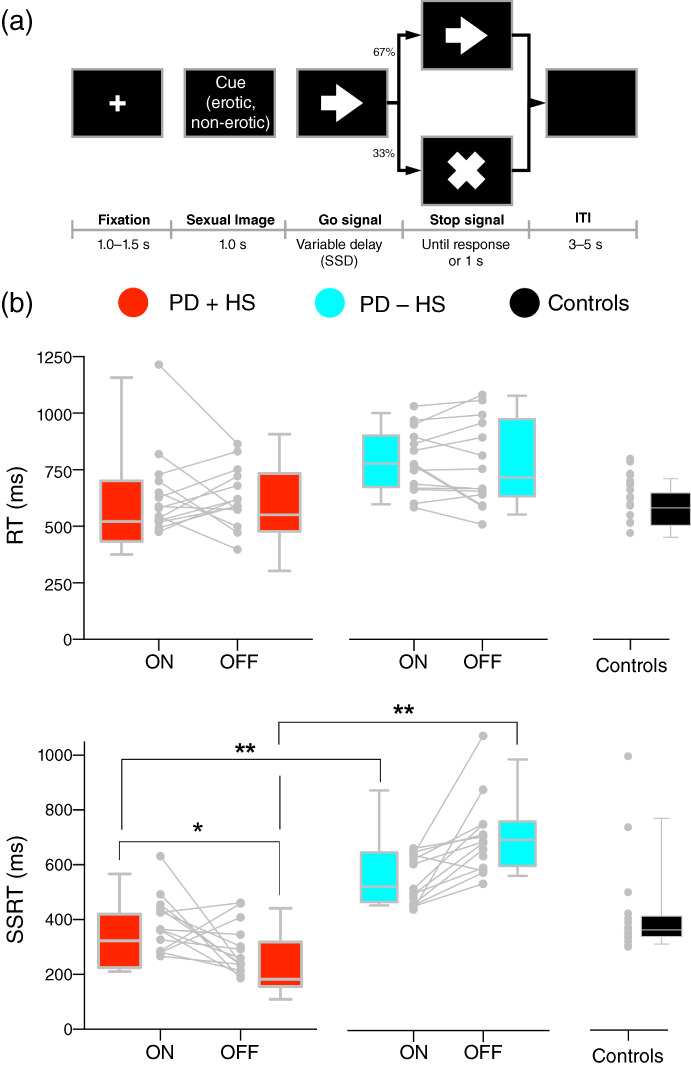
Behavioral paradigm and erotic stop‐signal results. (a) Task trial example. (b) Initiation response times (top) and stop‐signal reaction time (SSRT) (bottom) between medication (on vs off) and groups as well as individual values. Results are shown as box‐and‐whisker plots with each box representing the 10th to 90th percentiles where medians are represented by internal lines. ITI, intertrial interval; PD + HS, Parkinson disease with hypersexuality; PD − HS, Parkinson disease without hypersexuality; RT, reaction time; SSD, stop‐signal delay. *P < 0.05; **P < 0.01

The erotic stop‐signal task presented images of undressed (erotic) and dressed women (nonerotic). Left‐ or right‐pointing arrows (Fig. 1a) prompted participants to press using their index or middle finger of their right hand, respectively (limited time‐hold up to 1 s). Following some Go signals, stop‐signal cues (33%) were presented after a variable stop‐signal delay (staircase procedure using 50 ms steps), asking patients to stop their ongoing response. Null events during the intertrial interval ranged between 3 and 5 s. Twenty practice trials were performed before each study session. A total of 384 trials (192 per image condition) were presented per session (five blocks of 77 trials, study 1; three blocks of 128 trials, study 2). Response initiation, stop‐signal reaction time (SSRT; integration method by replacing Go omissions with the maximum reaction time values per participant^17^), stop‐signal delay, and errors were collected. Go trials above 3.5 standard deviations (SDs) from the participant's mean reaction time were discarded from the analysis. As previously recommended,^17^ we first evaluated the independence of the Go and Stop processes by: (i) correlations between StopRespond reaction times and correct Go trials of each stimulus condition (erotic and nonerotic) per participant; and (ii) cumulative distributions differences between Go and StopRespond reaction times (Data S1).

### Behavioral, neuropsychological, and neuropsychiatric tests

Measures of general cognition, executive functioning, sexual activity, and other neuropsychiatric variables related to ICD were collected during the on‐medication state. Further details can be accessed in the Appendix S1.

### Task‐based fMRI analysis

fMRI preprocessing steps are described in the Appendix S1. Task‐related activities were analyzed by sexual cue types using the following regressors: Go, Stop‐Inhibit, Stop‐Respond, null, and motion regressors, derivatives, and quadratic forms, representing each event with an impulse function convolved with a canonical hemodynamic response function. fMRI significance was considered at *P <* 0.005, with family‐wise error (FWE) correction at cluster level (*P <* 0.05). Small‐volume correction for anatomically defined regions of interest was conducted in the VTA based on a priori hypothesis that links this region with motivated behaviors.^17^

### Dynamic causal modeling

DCM^18^ (see Appendix S1) estimates the effective connectivity on 13 generative models representing alternative hypotheses of the causal interactions between cortical–subcortical areas guiding hypersexuality. Structural architecture of our models (Fig. S1) was informed by primate anatomical and human functional connectivity.^19^, ^20^, ^21^, ^22^, ^23^, ^24^, ^25^, ^26^ All models were constructed, fitted, and compared for each patient. Bayesian model selection^27^ was used to account for activity in the cortical and subcortical regions during the task and estimate the circuit abnormalities in hypersexuality.^27^ Effective connectivity strength and task modulations were compared with random‐effects Bayesian model averaging to obtain average connectivity estimates across models and patients.^27^

### DTI processing

Based on the DCM regions, we tested possible underlying structure alterations that sustain model differences between groups. For this we inspected the different fiber directions among them (Data S1). For tract selection across groups, automated fiber quantification^28^ was used to compute the fractional anisotropy and mean diffusivity over the tracts, ensuring that the approximate length and shape were similar between patients. A total of 100 nodes for fractional anisotropy values were obtained for each fiber bundle. To compute the difference between groups in fractional anisotropy and mean diffusivity, *t*‐tests over the nodes for every tract (using threshold‐free cluster‐enhancement [TFCE] as a statistical method) were performed between groups followed by nonparametric permutation tests (*P <* 0.05, FWE Holm‐Bonferroni corrections; using the code from ptsa^29^ and MNE^30^).

### Repetitive transcranial magnetic stimulation

In study 2, we used rTMS (Magstim Rapid^2^ Plus^1^ stimulator, Magstim) to apply intermittent theta‐burst stimulation^32^ over the pre‐SMA (*x* = 6, *y* = 24, *z* = 58; see Table 3, Stop‐Inhibit erotic vs nonerotic from study 1 results). This excitatory protocol consisted in delivering trains of 5‐Hz bursts for 2 s repeated every 10 s (20 cycles, 600 pulses). Each burst contained three pulses at 50 Hz. Stimulus intensity was set to 100% of the active motor threshold, determined by stimulating the dominant primary motor cortex area corresponding to the patients' first dorsal interosseous muscle. Scalp‐to‐cortex distance correction was used for intensity adjustments.^33^ Individual T1‐weighted structural MRI scans were loaded into a frameless stereotactic system (BrainSight, Rogue Research, Canada) and a Polaris infrared (NorthernDigital, Canada) was used to locate the target area. Registration to Montreal Neurological Institute (MNI) space was conducted after manual identification of the anterior–posterior commissure. The TMS coil was placed tangentially over the target area after adjusting for the patient's scalp anatomical landmarks to their own MRI scan. A control condition with sham stimulation was performed using a sham‐controlled coil (AirFilm Placebo, Magstim), controlling for device appearance and physical properties (i.e. sound) of stimulation.

**Table 3:** Stop‐Inhibit in erotic vs nonerotic trials

	*T*‐value	*X*	*Y*	*Z*
**Medication**				
Superior parietal lobe	4.02	−62	−8	−14*
	3.29	−56	4	−18*
	3.89	−52	−20	54*
**PD + HS on > off**				
Superior parietal lobe	4.04	−34	−6	64**
Middle frontal gyrus	3.67	−14	28	58**
Anterior cingulate cortex (dorsal)	3.62	10	26	46**
	3.53	4	28	20*
Caudate	3.50	−6	8	16*
	3.36	−14	2	14*
Ventral tegmental area	3.37	−2	−16	−12*,†
**PD + HS > PD − HS on**				
Superior parietal lobe	4.35	−52	−20	54**
	3.91	−36	2	60**
	3.84	−42	−26	46**
Pre‐supplementary motor area (pre‐SMA)	3.77	6	24	58**
	3.73	−14	30	58**
	3.66	−24	22	56**

*Note*: Tests and anatomical structures for the successful (Stop‐Inhibit) in erotic vs nonerotic trials. *P* < 0.05 family‐wise error cluster‐wise corrected.

Abbreviations: PD + HS, Parkinson disease with hypersexuality; PD − HS, Parkinson disease without hypersexuality; SMA, supplementary motor area.

* *P <* 0.005.

** *P* < 0.001.

† Small volume correction.

### Statistical analysis

Differences between conditions were analyzed using a mixed‐effects model comparing medication (on vs off) and condition (erotic vs nonerotic) as within‐subject and groups (PD + HS vs PD – HS vs controls) as the between‐group variable for successful and unsuccessful Stop trials. When comparing SSRT between medication (on vs off, study 1) and stimulation (real vs sham, study 2), support for the alternative hypothesis (H–: SSRToff < SSRT on) versus the null hypothesis (H0: SSRToff = SSRTon) was also assessed with Bayesian paired *t*‐tests (implemented in JASP version 0.14,^34^ with default effect size priors, Cauchy scale 0.707). Results are reported as one‐tailed Bayes factor (i.e. *BF*_−0_ represents [data|H−]/[data|H0]). Bonferroni correction for multiple comparisons were used in post hoc tests and specified when tests did not survive corrections. Moreover, correlations with neuropsychological and clinical variables were executed with response inhibition measures. Brain‐behavior Pearson correlations were executed between SSRT and beta weights extracted from brain regions of interests, fractional anisotropy, and mean diffusivity (mean values along the segments for each tract) and with effective connectivity.

## Results

### Clinical and demographic characteristics

In study 1, a total sample size of 14 PD + HS, 14 PD − HS, and 16 healthy controls were included in the final analysis (Table 1). First, patients were excluded because of significant reductions in hypersexuality between scanning sessions (*n* = 2 patients with PD + HS) and motion artifacts (*n* = 3 patients with PD − HS; *n* = 1 control). Second, a total of 18 patients with PD + HS were included in the final analysis in study 2 (exclusions due to reduced hypersexuality between sessions, *n* = 2). Groups were matched in terms of age, education, and clinical variables. Patients with PD + HS experienced other ICD manifestations, but hypersexuality was the most relevant according to the initial interview and scores on QUIP. As part of the recruitment process, two‐step interviews rejected seven patients (*n* = 1 depressed patient, *n* = 1 unwilling to spend time in the scanner, *n* = 3 cognitive deficits, and *n* = 2 homosexual patients). The high homogeneity and specificity of the sample turned recruitment challenging but adequate sample sizes were reached based on previous neuroimaging^35^, ^36^ and rTMS studies in PD and ICD.^37^

### Behavioral results

Our behavioral task generated enhanced arousal in patients with PD + HS while on medication (Table 2). Under the race‐stopping model, going and stopping are supposed to act independently,^37^ confirmed in our data by faster StopRespond reaction times compared with Go trials in every group (probability distributions test for Kolmogorov–Smirnov: all *P‐*values < 0.05 except for *P* = 0.06 for PD – HS off medication) (Fig. S4).

Response initiation (Go trials) revealed an effect of condition (Fig. 1b; *F*_(2, 44_) = 7.59, *P =* 0.009) and condition × group interaction (*F*_(2, 44_) = 4.45, *P =* 0.017). Post hoc comparisons of Go reaction times did not survive Bonferroni corrections. Sexual influence over response inhibition (SSRT) showed a triple interaction with medication, group, and condition (*F*_(2, 40)_ = 5.96, *P =* 0.005). Group and medication effects modulated response inhibition differently (Table S1; Fig. 1c; medication × group, *F*_(2, 44_) = 5.92, *P* = 0.006; group, *F*_(2, 44)_ = 7.07, *P* = 0.002), where post hoc tests showed significant SSRT increase in PD + HS attributable to medication (*t*_(12)_ = − 3.06, *P* = 0.01). Moreover, strong evidence was found on the impairment of response inhibition by sexual cues induced by medication in patients with PD + HS (*BF*_−0_ = 11.60). Similar differences were present between patient groups in nonerotic conditions when on (*t*_(27)_ = − 3.72, *P* = 0.001) and off medication (Table S1, *t*_(27)_ = − 6.57, *P* < 0.001). Performance while off medication explained the improved SSRT in patients with PD + HS, as shown by positive between SSRT on–off medication difference score and the on‐medication state (*r* = 0.753, *P* = 0.003). To test for specific effects of reactive inhibition, SSRT without influence of erotic cues revealed a group effect (*F*_(2, 44)_ = 7.13, *P* = 0.002) (Table S2) driven by prolonged SSRT in the PD − HS group compared with the PD + HS group (*t*_(27)_= − 3.07, *P* = 0.005) and controls (*t*_(28)_ = 3.70, *P* = 0.001). As expected, post hoc analyses revealed no impairments in patients with PD + HS compared with controls (*t*_(29)_= −0.09, *P* = 0.92).

## fMRI Results

### Motivational activation for erotic images

To establish the brain areas responding for sexual cues, a general linear model revealed a disease × medication interaction in a cluster along the cingulate cortex (anterior midcingulate; [0, 2, 38]). This cluster was more active in patients with PD + HS off > on medication and in patients with PD – HS on > PD + HS on medication.

### Neural network for inhibition under erotic influence

**Fig. 2:**
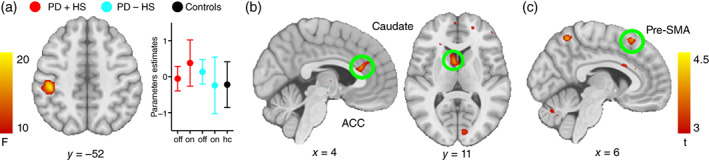
Neural correlates for successful inhibition in erotic vs nonerotic conditions in study 1. (a) Medication × group interaction with parameter estimates over superior parietal cortex per group. (b) *Medication* effect revealing increased activity of anterior cingulate cortex (ACC) and caudate head in patients with Parkinson disease with hypersexuality (PD + HS) comparing on‐ vs off‐medication states. (c) *Group* effect revealing enhanced activity of the pre‐supplementary motor area (pre‐SMA) in the PD + HS group compared with patients with PD without hypersexuality (PD – HS) (on‐medication).

To decipher group and medication effects on key brain areas behind successful inhibition (Stop‐Inhibit) under sexual influence, a general linear model on group, condition and medication revealed a medication effect in the superior parietal lobe (Table 3; Fig. 2a). This medication effect was driven by the on‐medication state in the PD + HS group also recruiting the ACC, middle frontal gyrus, caudate, and VTA (Table 3; Fig. 2b). Meanwhile, a group effect showed increased activity over the pre‐SMA in the PD + HS group compared with the PD – HS group (Table 3; Fig. 2c). Moreover, sexual influence on successful inhibition of actions (compared with unsuccessful inhibition) revealed a main effect of medication over the superior parietal lobe, ACC, and supplementary motor area (Table S3; Fig. S2a). This effect was mediated by the PD + HS group (on vs off medication) showing enhanced activity in the frontal pole, ACC, supplementary motor area, and inferior frontal gyrus (Table S3; Fig. S2b). The main effects of sexual influence over successful inhibition of actions (compared with Go trials) are detailed in the Supporting Information section (Table S4).

### Cortical–subcortical influences underlying response inhibition

**Fig. 3:**
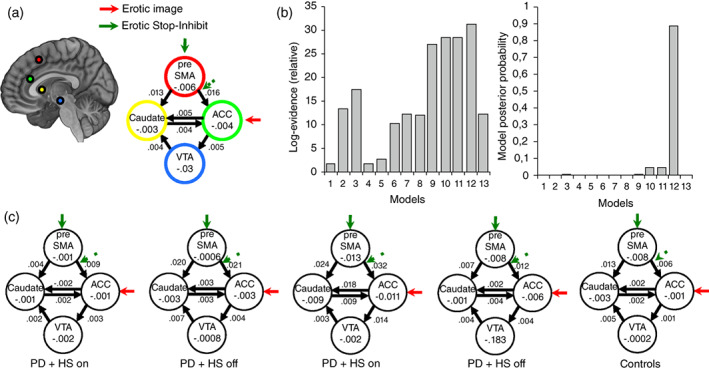
Dynamic causal modeling (DCM) results on the winning model and group differences. (a) Bayesian model selection at the group level, with dotted arrows indicating modulatory effects of stopping (correct Stop‐Inhibit) and erotic cues. Solid bold arrows indicate driving inputs of task performance locally. The most likely model counts with increased excitatory connectivity from the pre‐supplementary motor area (pre‐SMA) to the caudate and anterior cingulate cortex (ACC) (DCM.A average connectivity). This connectivity is modulated locally in pre‐SMA by task (DCM.B) and by modulatory inputs in descending connection towards ACC (DCM.A). Bidirectional connections between ACC and caudate are detected (DCM.A). (b) Using the free‐energy estimate of the log‐model evidence between models, very strong evidence was found in favor of model 12. The group posterior probability (close to 1) illustrates the strong support for the most likely model. (c) Model connectivity values after Bayesian parameter averaging at the group level reveal reduced excitatory connectivity in pre‐SMA to caudate pathway between patients with Parkinson disease with hypersexuality (PD + HS) and patients with Parkinson disease without hypersexuality (PD – HS). Ultimately, local task modulation over pre‐SMA differed between patients. VTA, ventral tegmental area.

Cortical–subcortical models comparison revealed striking results: there was strong evidence in favor of a model including the pre‐SMA to both caudate and ACC connections as excitatory (group average; Fig. 3a,b). Importantly, lower activity in pre–SMA‐caudate connectivity were seen when comparing patients with PD + HS (0.004 ± 0.006) versus patients with PD − HS (0.02 ± 0.03) while medicated (Fig. 3c; *P* = 0.02). Moreover, pre–SMA‐caudate connectivity was reduced while on medication (0.004 ± 0.006) compared with off medication (0.02 ± 0.05) in patients with PD + HS (Fig. 3c; *P =* 0.03). Local intrinsic connectivity in the caudate correlated with SSRT in patients with PD + HS while medicated (*r* = 0.55, *P =* 0.04), which reflects local neuronal adaptation indicative of an altered neurobiological substrate to explain response inhibition changes.

### Local modulatory effects of Stop‐Inhibit

The winning effective connectivity model revealed a task modulation (Stop‐Inhibit in erotic vs nonerotic) over pre‐SMA, which differed significantly between the PD + HS (0.04 ± 0.11) and PD − HS groups (Fig. 3c; −0.10 ± 0.16; *P =* 0.01). This finding is indicative that local pre‐SMA responses to Stop cues varies between patients and may contribute to descending aberrant signals that control behavior.

### Structural connectivity

**Fig. 4:**
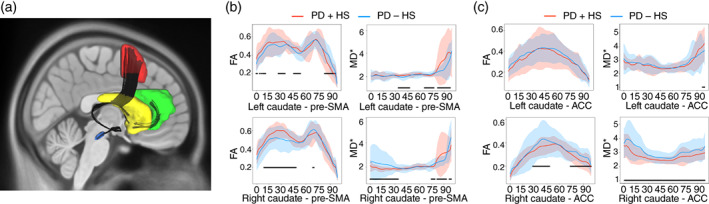
Tract representation and diffusion tensor imaging scalar measures—fractional anisotropy (FA) and mean diffusivity (MD). (a) Tract segmentation between areas in the dynamic causal modeling (ventral tegmental area, caudate, anterior cingulate cortex [ACC], pre‐supplementary motor area [pre‐SMA]) in the HCP1065 template, superposed on a T1 MNI template. (b) FA and MD along the segments of caudate and pre‐SMA tracts and (c) caudate and ACC. Significant differences (<0.05 corrected) along the segments between the groups are underlined in black. PD + HS, Parkinson disease with hypersexuality; PD − HS, Parkinson disease without hypersexuality.

Among the tract segmentation between the winning model regions (Fig. 4a), we observed bilateral significant differences in the pre–SMA‐caudate connection, showing higher fractional anisotropy values and lower mean diffusivity in patients with PD + HS compared with those with PD − HS (Fig. 4b **)** and controls (Fig. S3). Interestingly, the opposite pattern is seen for the caudate‐ACC connection with lower fractional anisotropy, but also reduced mean diffusivity values in patients with PD + HS compared with patients with PD − HS (Fig. 4c). The VTA‐caudate tract could not be reconstructed robustly with the DTI model and the proposed tractography algorithm, therefore it was excluded from the analysis. A relevant approaching‐significance negative correlation was found between fractional anisotropy and DCM from the right pre‐SMA and caudate connection in patients with PD + HS (*r* = 0.69; *P* = 0.06). Thus, higher fractional anisotropy in patients with PD + HS corresponds to lower effective connectivity, consistent with pathological compensatory responses to preserve functional connectivity.^39^, ^40^ Along the right pre–SMA‐caudate tract, mean diffusivity values showed a positive correlation with SSRT for patients with PD – HS (*r* = 0.79, *P =* 0.01) but a nonsignificant negative correlation in patients with PD + HS (*r* = −0.20; *P =* 0.61) during off medication. Thus, structural connectivity predicted behavioral changes between patient groups, confirming an altered anatomical connectivity behind the functional and behavioral disturbances in patients with PD + HS.

### Noninvasive brain stimulation: restoring control of hypersexual behavior

**Fig. 5:**
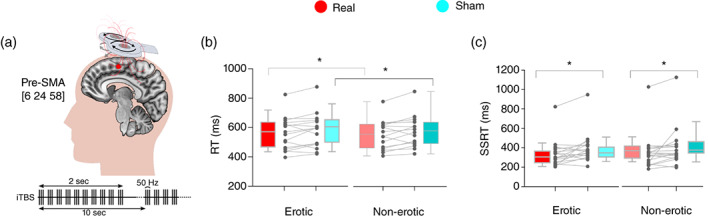
Neuromodulation effects on response inhibition and hypersexuality. (a) Cortical target location, coil position, and neuromodulation protocol description (adapted from Huang *et al*.^32^). (b) Initiation response times and (c) stop‐signal reaction time (SSRT) between sessions (real vs sham) as well as individual values during on medication. *P < 0.05. Results are shown as box‐and‐whisker plots with each box representing the 10th to 90th percentiles where medians are represented by internal lines. iTBS, intermittent theta‐burst stimulation; RT, reaction time; pre‐SMA, pre‐supplementary motor area.

Independence race‐model assumptions were also confirmed in study 2 (Fig. S4). Following real intermittent theta‐burst stimulation over the pre‐SMA (sham‐controlled), nonsignificantly faster initiation times were seen in erotic trials (*P =* 0.06) but were significant for nonerotic trials (Fig. 5b; *P =* 0.04). Importantly, significant improvement in response inhibition was found for erotic (*P =* 0.02) and nonerotic stimuli (Table S5; Fig. 5c; *P =* 0.04), together with moderate evidence towards the alternative hypothesis (*BF*−_0_ = 4.99). Correlation analysis between performance on the stop‐signal task and neuropsychological tests revealed that larger improvements in SSRT correlated with working memory (digit‐span test) after intermittent theta‐burst stimulation (*r* = 0.49; *P =* 0.03). Study 2 findings were not affected by possible session order effects (*F*'s > 1).

## Discussion

Here we show that response inhibition was diminished in patients with PD + HS when exposed to sexual cues comparing on vs off medication. The cortical‐striatal regions related to uncontrolled sexual behaviors included inhibitory regions such as the pre‐SMA, but also the VTA, caudate, and ACC as part of the limbic system. Effective connectivity models revealed reduced engagement of the pre‐SMA in stop‐related trials and reduced functional connectivity with the caudate in patients with PD + HS compared with patients with PD – HS1c. This functional change was accompanied by anatomical reshaping showing higher fractional anisotropy and reduced mean diffusivity in the pre–SMA‐caudate pathway in patients with PD + HS. Importantly, excitatory neuromodulation over the pre‐SMA improved response inhibition under sexual influence in patients with PD + HS. We provide compelling evidence to understand the neurobiological underpinnings of hypersexual behavior driven by medication in patients with PD + HS and describe a novel potential use for noninvasive neuromodulation to treat hypersexuality.

### Sexual cues impair response inhibition in PD + HS

We found greater disruption of response inhibition in the presence of sexual cues (Fig. 1b), an effect driven by dopaminergic medication. Using a classic stop‐signal task, prior mixed evidence shows no differences^40^ or proficient inhibition in ICD compared with patients with non‐ICD,^40^, ^41^ without observable differences between on–off medication regimens.^41^ Our findings using classic measures of reactive inhibition coincides with prior evidence^41^ but provides further specificity to the value of adding impulsivity‐related cues to test sexual disturbances over behavior. Indeed, the inhibitory network requires the integrity of inferior frontal gyrus, pre‐SMA, striatum, and the subthalamic nucleus^12^, ^13^, ^42^, ^43^, ^44^ to adequately function. Importantly, this network is altered in medicated patients with PD who have ICD.^45^, ^46^ Thus, unaffected response inhibition in patients with PD and ICD^40^, ^41^, ^47^ contrasts with altered engagement of the inhibitory network.^45^, ^46^ This controversy may be explained by the fact that most studies evaluate behavior without stimuli that boost patients' impulsive problems and include mixed ICD subtypes. Overall, we provide notions to update behavioral methods (using specific stimuli related to the clinical problem) to help solve current debates as to why some studies report unimpaired response inhibition in ICD^41^, ^47^ and advance fundamental knowledge of impulsive‐related disorders.

The motivational disturbance in patients with PD and ICD is sustained by greater tendency for novelty seeking,^48^ enhanced incentive salience,^35^ and reduced learning from negative outcomes,^49^ compatible with recent views on altered reward processing. Given that reward sensitivity increases in patients with PD while they are medicated,^50^ our results support a bias towards enhanced incentive salience that puts constraints on inhibitory behavior. The incentive salience hypothesis^51^ suggests that combinations of previously acquired cue–reward associations may put significant constraints on behavior. Since emotional inputs deactivate essential cognitive control regions,^52^ the presence of previously experienced sexual cues will downgrade top‐down control and permit uncontrolled sexual behavior to occur. Thus, prior reports that have shown intact cognitive control abilities in ICD^41^, ^47^ may be reframed as a net effect on inhibition, as no inputs from cue–reward associations were influencing patients' behavior. Our results are in line with both enhanced sensitivity to sexual cues together with reductions in response inhibition ability (observed while medicated) that altogether explain one form of impulsivity to sexual cues in ICD.

### Dysfunctional circuitry in hypersexuality: functional and anatomical changes

The circuitry mediating sexual events in healthy people engages limbic areas such as the ventral striatum, amygdala, ACC, and orbitofrontal cortex.^54^ Controlling sexual actions, in contrast, recruits specific regions of the inhibitory network, including the pre‐SMA, inferior‐frontal gyrus, superior parietal cortex, and ACC.^55^ Here, we found increased activity in the limbic circuitry (including ACC, VTA, and caudate) and pre‐SMA during response inhibition under sexual influence in patients with PD + HS (on vs off medication) compared with patients with PD − HS (Fig. 2c). We interpret these findings to be indicative of abnormal enhanced mesocorticolimbic activity without adequate control that may predispose to hypersexuality. With a faulty mesocorticolimbic circuitry mediated by dopaminergic drugs, including the VTA, ACC, and ventral striatum,^56^, ^57^ enhanced ‘wanting’ may thus lead to ‘hard‐to‐resist’ rewards.^58^ Hence, the combined wanting and hard‐to‐resist elements may integrate the neurobiological foundations of hypersexual behavior often found in patients with PD.^15^ Indeed, higher wanting and increased sexual desire was reported in patients with PD + HS after performance on the erotic stop‐signal task (Table 2), which may require larger ACC engagement and increase the wanting variant during uncontrolled behaviors. The ACC is well‐known to be operant during the early cycles of sexual behavior.^59^ Thus, a combination of dysfunctional dopaminergic bottom‐up (limbic) and top‐down (executive) control under medication may boost uncontrolled desire in our patients. This view matches well with the dual control model of sexual response,^60^ where a shared duality between sexual excitatory and inhibitory mechanisms regulates sexual behavior. The dual control model and plasticity alterations after persistent reward cue exposure^52^, ^61^ may predispose one of the two dual systems to set disproportionally high sexual excitation (driven by ACC sexual engagement) or disproportionally low sexual inhibition (driven by lost pre‐SMA functional control). Our results support the view that an enhanced sexual excitation system in the mesocorticolimbic system finds absent or reduced opposition from prefrontal top‐down control regions (namely pre‐SMA), leading to diminished control over sexual behavior.

The involvement of the above brain structures in ICD pathophysiology has been previously reported, but the way this network malfunctions to produce ICD is unknown. As revealed by a winning functional connectivity model (Fig. 3a), the pre‐SMA and its connectivity with the caudate put forward two pathophysiological assumptions in the hypersexual circuit: the pre‐SMA showed a reduced response to task‐related signals and weakened its excitatory connection with the caudate (Fig. 3c). We wanted to ascertain whether the top‐down dysfunctional connection (based on DCM) from pre‐SMA to caudate was a by‐product of underlying anatomical changes. Indeed, higher fractional anisotropy and lower mean diffusivity values were detected in the tracts of PD + HS group compared with the PD – HS group. Possibly, the combined change in functional and anatomical connectivity may both explain hypersexuality. Ultimately, opposite correlations between patients were seen between the pre–SMA‐caudate tract and SSRT (positive for patients with PD − HS while negative for patients with PD + HS), which overall suggests a dual neurobiological problem (local and descending pre‐SMA connectivity) in the inhibitory network.^62^, ^63^ Thus, reductions in top‐down control rely on paradoxical increases of white matter integrity, a possible neural compensatory response previously reported in patients with PD and ICD.^40^, ^64^, ^65^

The neurobiological changes in pre‐SMA to caudate connectivity in our PD + HS sample may be explained by experience‐dependent plasticity.^61^ Previous evidence indicates how repeated behaviors lead to experience‐dependent tract changes after daily training of executive function^66^ or after repeated alcohol consumption.^67^ Based on this evidence, we suggest that the compulsive repeated use of sexual acts may lead to frequent engagement and overflow activity in pre–SMA‐caudate connections. In a similar vein, pre‐SMA reductions to striatal subregions has been recently shown in patients with PD and ICD while performing a stop‐signal task.^47^ We speculate that aberrant plasticity in this pathway may increase the vulnerability to develop PD + HS and other forms of addictive behaviors. Together with notions that the mesocorticolimbic circuitry is overstimulated in PD and ICD,^2^, ^8^ the pre–SMA‐caudate tract may be a susceptible pathway to drug‐induced dopaminergic overstimulation and hypersexuality in PD, as seen in other addictions.^67^, ^68^

### A route towards therapy: cortical neuromodulation to control hypersexuality

To date, there is no directly validated treatment for hypersexuality. With the aim of improving this unfavorable clinical scenario, we compared excitatory rTMS stimulation over the pre‐SMA with a sham condition to reveal an acute benefit on response inhibition to sexual influence in patients with PD + HS (Fig. 5c). We favored stimulation of the pre‐SMA based on two foundations: its dominant role in response inhibition^43^, ^45^ and our fMRI group–based results from study 1. Based on previous reports of beneficial effects of rTMS on response inhibition^69^, ^70^, ^71^ and the excitability properties of intermittent theta‐burst stimulation over the pre‐SMA in inhibitory tasks,^71^ we assume our protocol enhanced pre‐SMA inhibitory function and hence control over sexual behavior. Similarly, changes in pre‐SMA to caudate connectivity may explain the behavioral change after neuromodulation due to their direct functional connection in response inhibition tasks.^62^ Also, intermittent theta‐burst stimulation may reset dysfunctional pre‐SMA downstream pathways and induce distant control over limbic networks in patients with PD + HS.

The mechanistic view of our neuromodulation findings is a possible change of the emotional regulation associated with sexual cues. Previously, a single‐blind, randomized, crossover study investigated the impact of pre‐SMA stimulation (online interference with five pulses at 10 Hz; sham‐controlled) on a facial emotion recognition task.^71^ After stimulation, a specific downregulation in recognition of happy faces was disrupted compared with sham stimulation.^71^ This finding directly involves the pre‐SMA in successful recognition of happy faces, suggestive of an influence on the reward system associated with human facial information. In line with such findings, our protocol could have produced an impact on human facial recognition in a sexual context in our patients and thus place fewer constraints over response inhibition. Yet, the modulatory effect of rTMS over response inhibition were unspecific as reductions in the SSRT were seen in both erotic and nonerotic trials, possibly attributable to general improvements in the response inhibition mechanism. Response inhibition is commonly affected in disorders where impulsivity is a hallmark.^72^ In fact, SSRT is a valuable predictor of recovery in substance abuse.^73^, ^74^ SSRT can be modulated by deep‐brain stimulation of the subthalamic nucleus in PD showing impairments,^75^ a surgical procedure linked to behavioral disinhibition in treated patients.^76^ SSRT improvements are accompanied by a reduction of symptoms in patients with attention‐deficit disorder treated with methylphenidate.^77^ Future studies using pre‐SMA neuromodulation could induce long‐lasting effects over response inhibition and result in significant clinical improvements in ICD and other conditions marked by pathological impulsivity.

### Strengths and limitations

One strength of this study was using a highly selected clinical sample and provocative stimuli to boost impulsivity in patients with PD + HS measured with multilevel neuroimaging tools. We then used a circuit‐based approach to stimulate a putative brain target essential in PD + HS. However, our findings are based on a moderate sample size that should be expanded to larger cohorts. The reasons for this sample size are directly linked to difficulties in recruitment. Yet, both studies provide adequate sample sizes based on previous neuroimaging^35^, ^36^ and rTMS studies in PD and ICD.^37^ Moreover, our Bayesian analysis shows increasing support for the alternative hypothesis, with strong and moderate evidence in studies 1 and 2, respectively.

There are several limitations to this work. Stimulation of pre‐SMA was conducted in patients with PD + HS, but patients without hypersexuality were not studied. Thus, the choice of this design renders our results not specific to hypersexual issues. Yet, using sham‐controlled stimulation provides one required form of control of our neuromodulation target and protocol. Moreover, improvements seen in the erotic stop‐signal task might be a nonspecific effect. Because of design and time constraints, we did not include other tasks or formal impulsivity assessments after rTMS to test for the specificity of this effect in response inhibition or acute clinical improvements.

## Conclusion

Evidence for an incentive sensitization framework is provided to explain the interplay between desire enhancement and response inhibition in hypersexuality. We reveal a circuit‐based route that deciphers the cortical‐striatal anatomical and functional dysfunction and a candidate brain target for neuromodulation to change response inhibition in patients with PD + HS.

## Disclosures

C.A. received grants from Juan de La Cierva (IJC2020‐045437‐I). R.M.F. received grants from Instituto de Salud Carlos III, honoraria for presentations from Insightec, Zambon, Bial, Palex and Boston Scientifics; and travel support from Insightec, Palex and Zambon. L.V.D. received travel support from Abbvie, Krka and Bial. F.A.F received honoraria for presentations from Bial and Abbvie and travel support from Zambon. Remaining authors disclose no conflict of interest.

## Funding information

Funding included Fundación Jesús de Gangoiti Barrera (D.M.M.), AES‐ISCIII‐Miguel Servet (CP18/00038) and AES‐ISCIII (PI19/00298) (I.O.).

## Author contribution

D.M.M. and J.A.P.P.: data collection, analysis, interpretation, and article writing/editing. M.M: data analysis, article writing/editing. P.C. and A.C.: data collection; R.M.F., J.A.M., L.V.D., and F.A.F: recruitment, clinical assessment and article editing. O.I.: Responsible design, data collection, analysis, interpretation, and writing/editing of the article.

## Ethics statement

The research follows the Declaration of Helsinki and was granted approval by the ethics committee of HM Hospitales (17.03.0852E2‐GHM).

## Informed consent

All participants gave written informed consent.

## Supporting information


**Appendix S1.** Supporting Information.
**Figure S1.** Structure of the 13 dynamic causal modeling (DCM) tested and selection of most likely model.
**Figure S2.** Successful vs unsuccessful inhibition activity maps.
**Figure S3.** Tracts representation and diffusion tensor imaging (DTI) scalar measures (fractional anisotropy [FA], mean diffusivity [MD]) for controls.
**Figure S4.** Density distribution for StopRespond and Go conditions. (A) Density distribution for StopRespond (white distribution) and Go conditions (filled distribution) for Parkinson disease with hypersexuality (PD + HS) and Parkinson disease without hypersexuality (PD – HS) on and off medication as well as for healthy controls. (B) Density distribution for StopRespond (white distribution) and Go trials (filled distribution) per stimulation session (real or sham) and per trial type (erotic or nonerotic).
**Table S1.** Erotic stop‐signal reaction time results per *Medication* and *Group* conditions
**Table S2.** Stop‐signal reaction time results per group
**Table S3.** Successful vs unsuccessful inhibition under erotic condition
**Table S4.** Successful Inhibition vs Go trials in the erotic condition
**Table S5.** Behavioral results of the stop‐signal task (study 2) after real and sham intermittent theta‐burst stimulation

## Data Availability

Codes and data analysis pipelines can be found in https://github.com/cinac-cognition/tms_fmri_dti_hypersexual_pd. Material and Correspondence Address all requests to Dr. Ignacio Obeso.
