# Supplementary material for: A circuit‐based approach to modulate hypersexuality in Parkinson's disease
Source: Psychiatry Clin Neurosci. 2023 Feb 3;77(4):223–32. doi: 10.1111/pcn.13523 (PMC11488615; doi:10.1111/pcn.13523)
Supplement: Supplementary file 1 — Appendix S1. Supporting Information. Figure S1. Structure of the 13 dynamic causal modeling (DCM) tested and selection of most likely model. Figure S2. Successful vs unsuccessful inhibition activity maps. Figure S3. Tracts representation and diffusion tensor imaging (DTI) scalar measures (fractional anisotropy [FA], mean diffusivity [MD]) for controls. Figure S4. Density distribution for StopRespond and Go conditions. (A) Density distribution for StopRespond (white distribution) and Go conditions (filled distribution) for Parkinson disease with hypersexuality (PD + HS) and Parkinson disease without hypersexuality (PD – HS) on and off medication as well as for healthy controls. (B) Density distribution for StopRespond (white distribution) and Go trials (filled distribution) per stimulation session (real or sham) and per trial type (erotic or nonerotic). Table S1. Erotic stop‐signal reaction time results per Medication and Group conditions Table S2. Stop‐signal reaction time results per group Table S3. Successful vs unsuccessful inhibition under erotic condition Table S4. Successful Inhibition vs Go trials in the erotic condition Table S5. Behavioral results of the stop‐signal task (study 2) after real and sham intermittent theta‐burst stimulation [file PCN-77-223-s001.docx]

**Supporting Information**

**A circuit-based approach to modulate hypersexuality in Parkinson’s disease**

David Mata-Marín^1,2,6^^, José A. Pineda-Pardo^1,2^^, Mario Michiels^1,2,6^, Cristina Pagge^1,2,6^, Claudia Ammann^1,2^, Raúl Martínez-Fernández^1,2^, José Antonio Molina^3^, Lydia Vela^4^, Fernando Alonso-Frech^5^, Ignacio Obeso^1,2,7*^

^Co-first authors

**Author affiliations:**

1 HM CINAC (Centro Integral de Neurociencias Abarca Campal). Hospital Universitario HM Puerta del Sur. HM Hospitales. Madrid, Spain.

2 Network Center for Biomedical Research on Neurodegenerative Diseases (CIBERNED), Instituto Carlos III, Madrid, Spain.

3 Hospital 12 de Octubre, Madrid, Spain.

4 Hospital Fundación Alcorcón, Madrid, Spain.

5 Hospital Clínico San Carlos, Madrid, Spain.

6 PhD program in Neuroscience, Autonoma University of Madrid, 28029 Madrid, Spain.

7 Department of Psychobiology & Methods for the Behavioral Sciences Department, Complutense University of Madrid, Madrid, Spain.

***Correspondence to:** Dr. Ignacio Obeso.

Avda. Carlos V, 70 Móstoles, 28938, Madrid, Spain.

[i.obesomartin@gmail.com](mailto:i.obesomartin@gmail.com)

[www.controlandhabit.com](http://www.controlandhabit.com/)

**Running title**: Hypersexuality alters cognitive control

# Methods Internal arousal state

Explicit measurements of sexual desire were collected in study 1 before and after task performance by asking participants to mark between 0 (nothing) to 10 (very much) on how intense their internal state for pre-scanning was:

1. How much sexual appetite did you experienced in the last hour?
2. Did you feel increased libido in the last hour?

Similarly, after the fMRI session, in addition to the first 2 items above, the following items were included:

3. How much you felt nude images corrupted or slowed your responses?
4. How much you felt dressed images corrupted or slowed your responses?

**Behavioural, neuropsychological and neuropsychiatric tests**

General cognition and executive measures were included to discard any cognitive impairments in our sample (Montreal Cognitive Assessment^1^, the Stroop interference test, Frontal Assessment Battery^2^, forward and backward digit span from Wechsler Adult Intelligence Scale IV^3^). Neuropsychiatric variables that may influence ICDs were included (Barratt Impulsiveness Scale-11^4^; Geriatric Depression Scale^5^; Starkstein Apathy Scale^6^; and Beck Anxiety Scale^7^). Last, a sexual inventory test (Brief Male Sexual Function Inventory^8^) was used to include the latest sexual activities in our sample.

**Imaging data acquisition**

Imaging data was collected using a 3T hybrid PET-MRI scanner (mMR Biograph, Siemens AG, Germany) with a 12-channel head array coil. Task-based fMRI was acquired using a single-shot gradient-echo echo-planar imaging (EPI) 2D pulse sequence with the following parameters: *TR/TE* = 2000/30ms; optimum flip angle using the Ernst equation i.e., 79°, *spatial resolution* = 3x3x4mm3; *field of view* = 192mm; *matrix* = 64×64; *slice thickness*= 4mm and acceleration factor of 2 (IPAT2). Five fMRI runs were acquired per session (260 volumes per run, 12mins) with 2-min pause between runs. Imaging protocol also included a 3D T1-weighted MP-RAGE (*TR/TE/TI* = 2300/3.34/900ms;*flip angle* = 8; and *isotropic spatial resolution* = 1mm3, *FoV* = 256mm, *matrix* = 256×256, *slice thickness* = 1mm); a field map generated from two 2D gradient-echo images (*TR/TE1/TE2* = 455/4.92/7.38ms, *flip angle* = 60° with the spatial resolution as the fMRI EPI volumes); and diffusion weighted images (DWI) using a single-shot 2D spin-echo EPI sequence (*TR/TE* = 10000/102ms; *isotropic resolution =* 2mm). DWI were acquired for 64 non-collinear encoding directions with *b-value*=1000s/mm2 and two *b*=0s/mm2 images with opposite polarity of the phase encoding direction (AP and PA).

fMRI preprocessing

fMRI preprocessing was carried out with tools from FSL (FMRIB Software Library) and SPM12. T1-weighted anatomical images for all subjects were segmented into gray matter, white matter, and cerebrospinal fluid using the unified segmentation tool^9^. Gray matter maps were then transformed into the Montreal Neurological Institute (MNI) space using the DARTEL tool^10^. Preprocessing included slice timing correction, motion correction by realigning to the first volume, correction of magnetic field inhomogeneity induced geometrical distortions and signal dropout, and co-registration to the anatomical images. Then, fMRI volumes were normalized to MNI space and smoothed with an 8mm3 FWHM Gaussian kernel and filtered over time using a high-pass filter of 128s. Omission errors and presses during sexual cues were classified as errors for the fMRI analysis.

Dynamic causal modelling (DCM)

To extract regional time series for dynamic causal modelling, we specified an F test across all trials (*F* contrast: *P<*.05) to obtain the first eigenvariate of the brain-oxygen-level dependent imaging time series from 4 volumes of interest in Stop-Inhibit erotic vs. non-erotic contrast: pre-SMA [*x*=6, *y*=24, *z*=58], ACC [*x*=0, *y*=24, *z*=36], caudate [*x*=-10, *y*=8, *z*=14], and VTA [*x*=-4, *y*=-16, *z*=-14]. Each subject’s *F* test was used to identify local maxima closest to the group peak to then extract the first eigenvariate from a 5mm sphere at the subject-specific peak.

DCM estimates the effective connectivity between brain regions according to (i) average connections between regions (DCM.A), (ii) modulatory task influence on connections (erotic images and Stop-Inhibit in erotic trials, see **Fig. 3A**; DCM.B) and (iii) condition-specific inputs that drive network activity (namely regional engagement in a Stop-Inhibit task; DCM.C). Across all 13 models, driving inputs (DCM.C) represented 2 key elements of the task: erotic images and Stop-Inhibit tasks. This was set to both ACC and pre-SMA areas (**Supplementary Fig. 1**). Bayesian parameter averaging and was used to estimate the connectivity values of the most likely model at the group level^11^.

DWI preprocessing steps

Preprocessing for DWI data was performed using QSIPrep0.13.0RC1, which is based on Nipype 1.6.0 (REFs 60 and 61) . The following preprocessing steps were conducted: Marchenko-Pastur distribution principal component analysis (MP-PCA) denoising following MRtrix3’s dwidenoise^12^ was applied with a 5-voxel window. After MP-PCA, B1 field inhomogeneity was corrected using dwibiascorrect from MRtrix3 with the N4 algorithm^13^. After B1 bias correction, the mean intensity of the DWI series was adjusted so all the mean intensity of the *b*=0 images matched across each separate DWI scanning sequence. FSL (version 6.0.3:b862cdd5) eddy was used for head motion correction and Eddy current correction^14^. Eddy was configured with a *q*-space smoothing factor of 10, a total of 5 iterations, and 1000 voxels used to estimate hyperparameters. A linear first level model and a linear second level model were used to characterize Eddy current-related spatial distortion. *q*-space coordinates were forcefully assigned to shells. Field offset was attempted to be separated from subject movement. Shells were aligned post-eddy. Eddy’s outlier replacement was run^15^. Data were grouped by slice, only including values from slices determined to contain at least 250 intracerebral voxels. Groups deviating by more than 4 standard deviations from the prediction had their data replaced with imputed values. *b*=0 reference image was collected with reversed phase-encode blips, resulting in a pair of images with distortions going in opposite directions. Here, *b*=0 images with reversed phase encoding directions were used along with an equal number of *b*=0 images extracted from the DWI scans. From these pairs the susceptibility-induced off-resonance field was estimated^16^. The fieldmaps were ultimately incorporated into the Eddy current and head motion correction interpolation. Final interpolation was performed using the jac method.

Several confounding time-series were calculated based on the preprocessed DWI: framewise displacement (FD) using the implementation in *Nipype* (following the definitions by Power *et al.*^17^) The head-motion estimates calculated in the correction step were also placed within the corresponding confounds file. Slice-wise cross correlation was also calculated. The DWI time-series were resampled to ACPC, generating a preprocessed DWI run in ACPC space with 1.2mm isotropic voxels. Several internal operations of *QSIPrep* use *Nilearn* 0.7.0^18^ (RRID:SCR_001362) and *Dipy*^19^.

DTI preprocessing and tract selection

Using the HCP1065 atlas, we ensured that diffusion tensor imaging was a viable model for this study and confirmed tracts do not have a significant number of crossing fibers. Diffusion tensor estimation was carried out using the non-linear least-squares method implemented by Dipy^19^. Spatial normalization in a tensor-based manner was done using dti-tk^20^. Then, tensors were normalized to a population template constructed by averaging each group of subjects. Finally, the images were mapped to 1mm^3^ MNI space. Relevant features – fractional anisotropy and mean diffusivity – were extracted at this point using dti-tk. A deterministic tracking algorithm was implemented in trackvis^21^, which resulted in whole brain tractography per subject.

We ran the default fiber tracking algorithm using DsiStudio^22^ with 1000000 seeds to select the tracts of interest (HCP1065 atlas). Based on the DCM results, we selected ROIs extracted from the AAL_2mm atlas (except for VTA: AAL_1mm) and the pre-SMA (HMAT 2mm atlas)^23^. Tracts were obtained by setting the first region as seed (e.g., caudate) and the end region as end (e.g., pre-SMA). However, the right caudate to ACC tract were both set as ROI to obtain a higher number of streamlines.

Then, streamlines were cut to avoid limiting access into the seed region. Finally, we deleted residual short tracts produced by cutting the tracts outside the seed region by setting the threshold to 10mm. For left caudate-ACC, we additionally deleted repeated tracts using the corresponding DsiStudio function (*voxel threshold* = 1). For right VTA-caudate, the straight streamline was removed since the tracking algorithm was not able to recognize the tracts streamlines from one of the hemispheres.

These bundles were used as a reference to extract the corresponding bundles from each subject. For this, we set to 20 the number of points to define each streamline and then we ran the recoBundles method implemented by Dipy with the default options.

# Results

Behavioural results

While *p*(inhib) did not significantly differ between groups [*F*’s>1], it varied from 50% (**Supplementary Table 1**) thus using the integration SSRT method is recommended^24^. Independence between going and stopping on erotic trials was confirmed with non-significant correlations between both measures (go and StopRespond RT) across groups [*P*’s>.05]. Also, faster StopRespond RT compared to Go RT independence between go and stop are all in line with a race model of action cancellation. Cumulative distributions differences between Go and StopRespond reaction times are displayed in **Supplementary Fig. 4A**. Mean stop signal delay (SSD) across groups did not vary significantly between groups nor between conditions., nor it revealed significant interactions [**Supplementary Table 1**; *F*’s>1].

Medication effects SSRT across conditions revealed an specific worsening of inhibition in PD+HS patients while on compared to off medication [**Supplementary Table 1**, *t*_(12)_=-3.06, *P=*.01], an effect not seen in the non-erotic condition [**Supplementary Table 1**, *t’s*<.1; *P<*.8]. Error rates were not significantly different across groups, medication, or condition [**Supplementary Table 1**, *z*’s<1]. Significant difference in response adaptation on the response delay effect (RDE) was seen (Go erotic minus Go non-erotic RTs) in the PD+HS group between medication states [*on*: -0.22±11.9; *off*: 13.87±17.48; *P<*.03], while non-significant in the PD-HS group [*on*: -5.40±2.3; *off*: 0.84±14.44; *P<*.35].

fMRI results

Neural Network for successful inhibition under erotic influence

To eliminate confounding factors of the motor response embedded within inhibition of ongoing actions, Stop-Inhibit vs. Go trials were compared showing a main Group effect, with significant clusters over the motor cortex and cerebellum (**Supplementary Table 4**). Such effect was driven by the PD-HS group showing greater ACC activity than PD+HS hypersexual patients (off medication; **Supplementary Table 4**).

Non-invasive brain stimulation: aiming to modulate hypersexual behaviour

Probability of inhibition substantially deviated from 50% in both real and sham (~70%; **Supplementary** **Table 5**), thus we used the integrative approach to estimate SSRT values No significant differences in SSD were observed in any type of stimuli after either condition (**Supplementary Table 5**). Independence between going and stopping on erotic trials was confirmed with non-significant correlations between both measures (go and StopRespond RT) across conditions [*P*’s>.05]. Also, faster StopRespond RT compared to Go RT independence between go and stop are all in line with a race model of action cancellation. Cumulative distributions differences between Go and StopRespond reaction times are displayed in **Supplementary Fig. 4B**.

Although not statistically significant, after real intermittent theta burst stimulation (compared to sham) patients produced less premature errors – anticipations during the sexual cue– in both erotic and non-erotic stimuli and tended to produce faster RTs in go trials (**Supplementary Table 5**). No significant difference in response adaptation on the response delay effect (*RDE*: Go erotic minus Go non-erotic RTs) was seen in any condition [*real*: 12.01±21.08; *sham*: 9.42±17.48; *P=*.90].

Supplementary references

1. Nasreddine ZS, Phillips NA, Bédirian V, et al. The Montreal Cognitive Assessment, MoCA: A brief screening tool for mild cognitive impairment. *J Am Geriatr Soc*. Published online 2005. doi:10.1111/j.1532-5415.2005.53221.x

2. Dubois B, Slachevsky A, Litvan I. The FAB. *Neurology*. Published online 2000. doi:10.1212/WNL.57.3.565

3. Weschler D. Wechsler Adult Intelligence Scale - Fourth Edition. *Stat Solut*. Published online 2008. doi:10.1080/00268978500101581

4. Patton JH, Stanford MS, Barratt ES. Factor structure of the Barratt impulsiveness scale. *J Clin Psychol*. 1995;51(6):768-774.

5. Yesavage JA, Brink TL, Rose TL, et al. Development and validation of a geriatric depression screening scale: A preliminary report. *J Psychiatr Res*. 1982;17(1):37-49. doi:10.1016/0022-3956(82)90033-4

6. Starkstein SE, Mayberg HS, Preziosi TJ, Andrezejewski P, Leiguarda R, Robinson RG. Reliability, validity, and clinical correlates of apathy in Parkinson’s disease. *J Neuropsychiatry Clin Neurosci*. 1992;4(2):134-139. doi:10.1176/JNP.4.2.134

7. Beck AT, Epstein N, Brown G, Steer RA. An Inventory for Measuring Clinical Anxiety: Psychometric Properties. *J Consult Clin Psychol*. 1988;56(6):893-897. doi:10.1037/0022-006X.56.6.893

8. Mykletun A, Dahl AA, O’Leary MP, Fosså SD. Assessment of male sexual function by the Brief Sexual Function Inventory. *BJU Int*. 2006;97(2):316-323. doi:10.1111/j.1464-410X.2005.05904.x

9. Ashburner J, Friston KJ. Unified segmentation. *Neuroimage*. 2005;26(3):839-851. doi:10.1016/j.neuroimage.2005.02.018

10. Ashburner J. A fast diffeomorphic image registration algorithm. *Neuroimage*. Published online 2007. doi:10.1016/j.neuroimage.2007.07.007

11. Stephan KE, Penny WD, Daunizeau J, Moran RJ, Friston KJ. Bayesian model selection for group studies. *Neuroimage*. 2009;46(4):1004-1017. doi:10.1016/j.neuroimage.2009.03.025

12. Veraart J, Novikov DS, Christiaens D, Ades-aron B, Sijbers J, Fieremans E. Denoising of diffusion MRI using random matrix theory. *Neuroimage*. 2016;142:394-406. doi:10.1016/j.neuroimage.2016.08.016

13. Tustison NJ, Avants BB, Cook PA, et al. N4ITK: Improved N3 bias correction. *IEEE Trans Med Imaging*. 2010;29(6):1310-1320. doi:10.1109/TMI.2010.2046908

14. Andersson JLR, Sotiropoulos SN. An integrated approach to correction for off-resonance effects and subject movement in diffusion MR imaging. *Neuroimage*. 2016;125:1063-1078. doi:10.1016/j.neuroimage.2015.10.019

15. Andersson JLR, Graham MS, Zsoldos E, Sotiropoulos SN. Incorporating outlier detection and replacement into a non-parametric framework for movement and distortion correction of diffusion MR images. *Neuroimage*. 2016;141:556-572. doi:10.1016/j.neuroimage.2016.06.058

16. Andersson JLR, Skare S, Ashburner J. How to correct susceptibility distortions in spin-echo echo-planar images: Application to diffusion tensor imaging. *Neuroimage*. 2003;20(2):870-888. doi:10.1016/S1053-8119(03)00336-7

17. Power JD, Mitra A, Laumann TO, Snyder AZ, Schlaggar BL, Petersen SE. Methods to detect, characterize, and remove motion artifact in resting state fMRI. *Neuroimage*. 2014;84:320-341. doi:10.1016/j.neuroimage.2013.08.048

18. Abraham A, Pedregosa F, Eickenberg M, et al. Machine learning for neuroimaging with scikit-learn. *Front Neuroinform*. 2014;8(FEB):1-10. doi:10.3389/fninf.2014.00014

19. Garyfallidis E, Brett M, Amirbekian B, et al. Dipy, a library for the analysis of diffusion MRI data. *Front Neuroinform*. 2014;8:8.

20. Zhang H, Yushkevich PA, Alexander DC, Gee JC. Deformable registration of diffusion tensor MR images with explicit orientation optimization. *Med Image Anal*. 2006;10(5):764-785. doi:10.1016/j.media.2006.06.004

21. Wang R, Benner T, Sorensen AG, Wedeen VJ. Diffusion Toolkit: A Software Package for Diffusion Imaging Data Processing and Tractography. *Proc Intl Soc Mag Reson Med*. 2007;15:3720.

22. Yeh F. DSI Studio. Published online June 2021. doi:10.5281/ZENODO.4978980

23. Mayka MA, Corcos DM, Leurgans SE, Vaillancourt DE. Three-dimensional locations and boundaries of motor and premotor cortices as defined by functional brain imaging: A meta-analysis. *Neuroimage*. 2006;31(4):1453-1474. doi:10.1016/j.neuroimage.2006.02.004

24. Verbruggen F, Aron AR, Band GPH, et al. A consensus guide to capturing the ability to inhibit actions and impulsive behaviors in the stop-signal task. *Elife*. 2019;8. doi:10.7554/eLife.46323

# Supplementary Figures

**Supplementary Fig. 1. Structure of the 13 DCMs tested and selection of most likely model.**


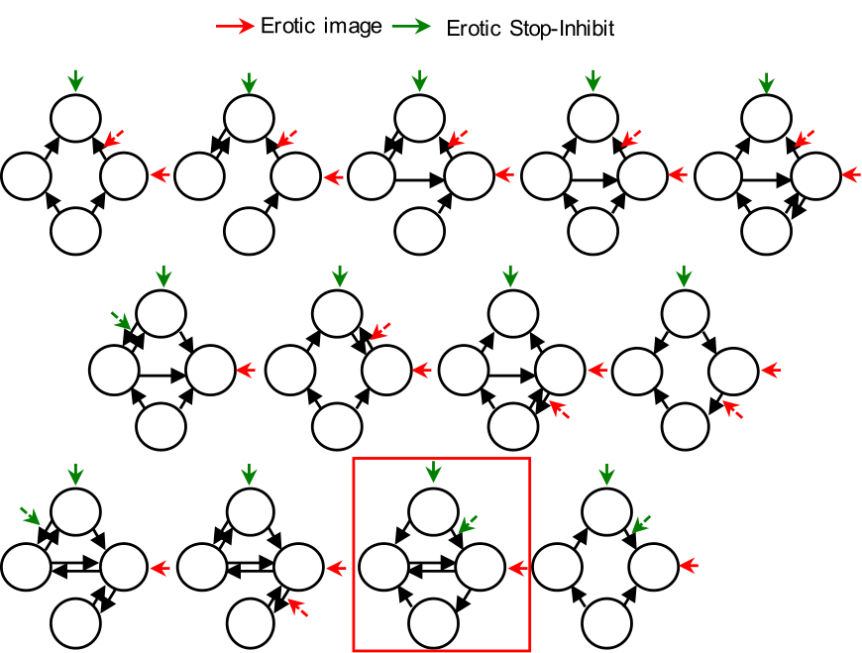


The 13 generative models represent alternative hypotheses of cortico-subcortical interactions during cognitive control while exposed to sexual cues in hypersexuality. The most likely model after Bayesian model selection (BMS) is highlighted in red square.

**Supplementary Fig. 2. Successful vs. unsuccessful inhibition activity maps.**


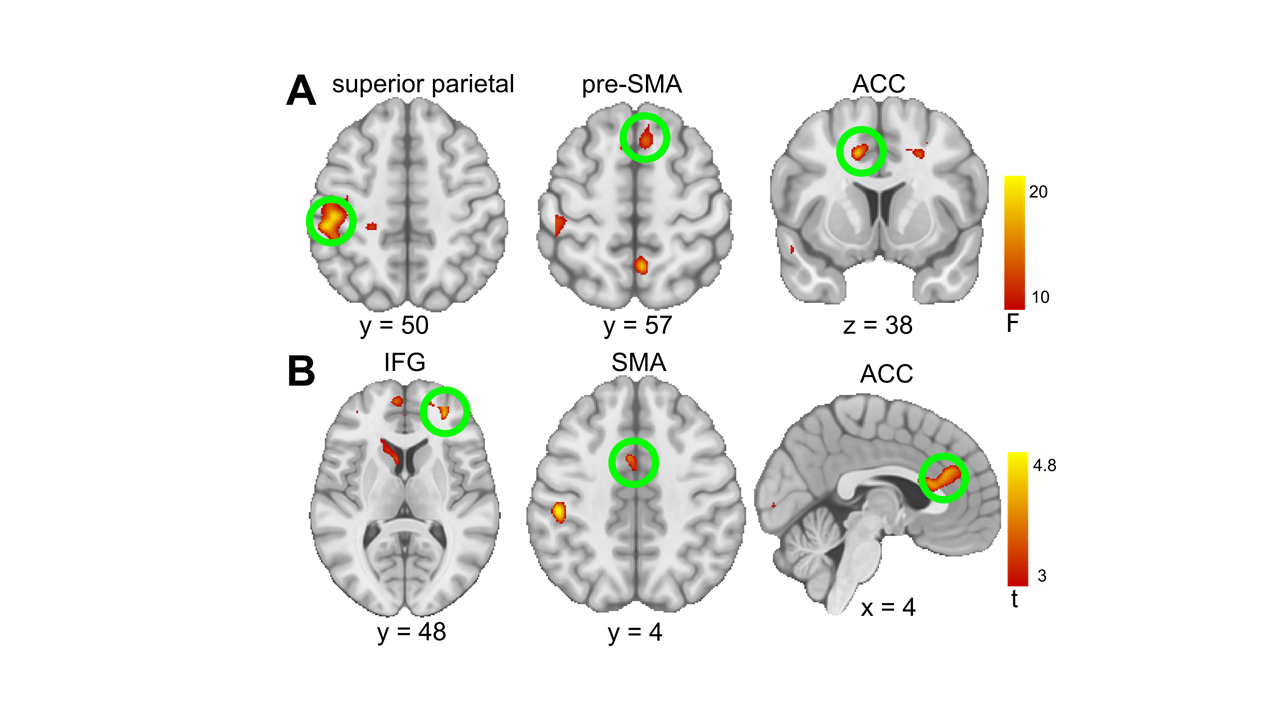


**(A)** Main effect of medication and **(B)** activity driven by PD+HS patients (on vs off medication). *ACC*: anterior cingulate cortex; *IFG*: Inferior-frontal gyrus.*;* *pre-SMA:* pre-supplementary motor area; *SMA*: supplementary motor area.

**Supplementary Fig. 3. Tracts representation and DTI scalar measures (FA, MD) for controls.**


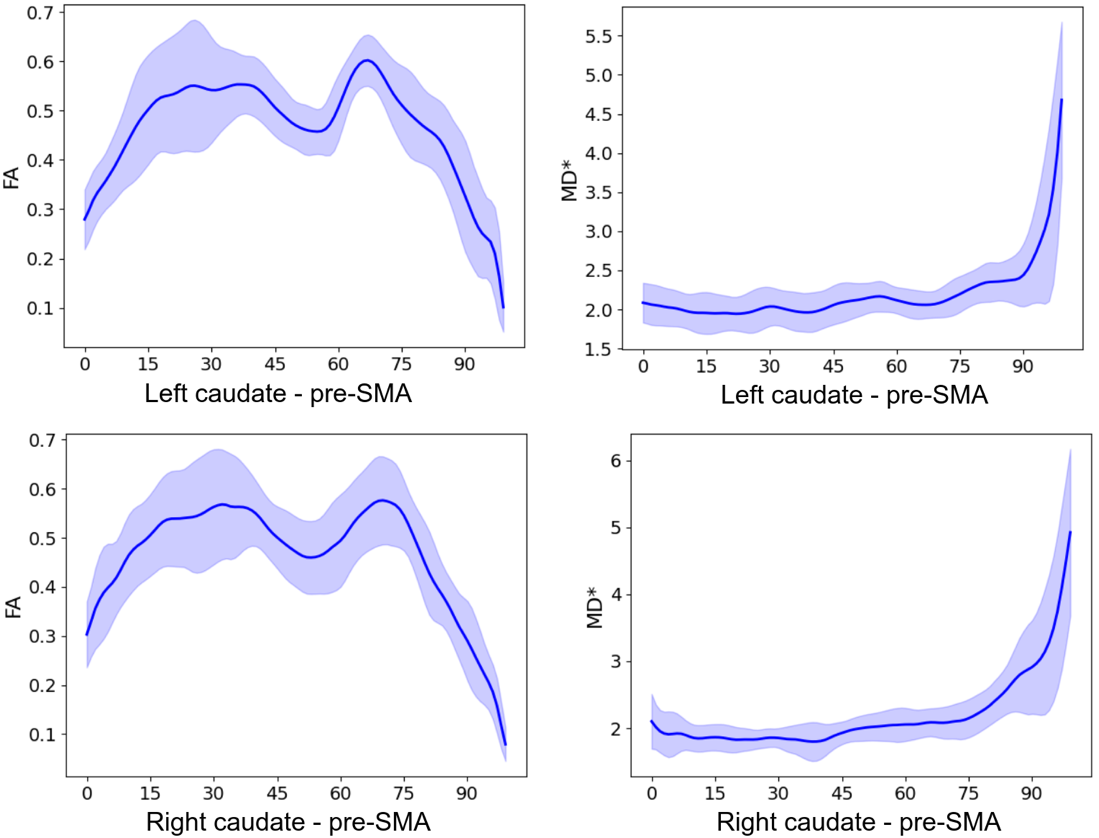


FA and MD along the segments of right-left caudate and pre-SMA tracts. *FA*: fractional anisotropy; *MD:* mean diffusivity*; pre-SMA:* pre-supplementary motor area.


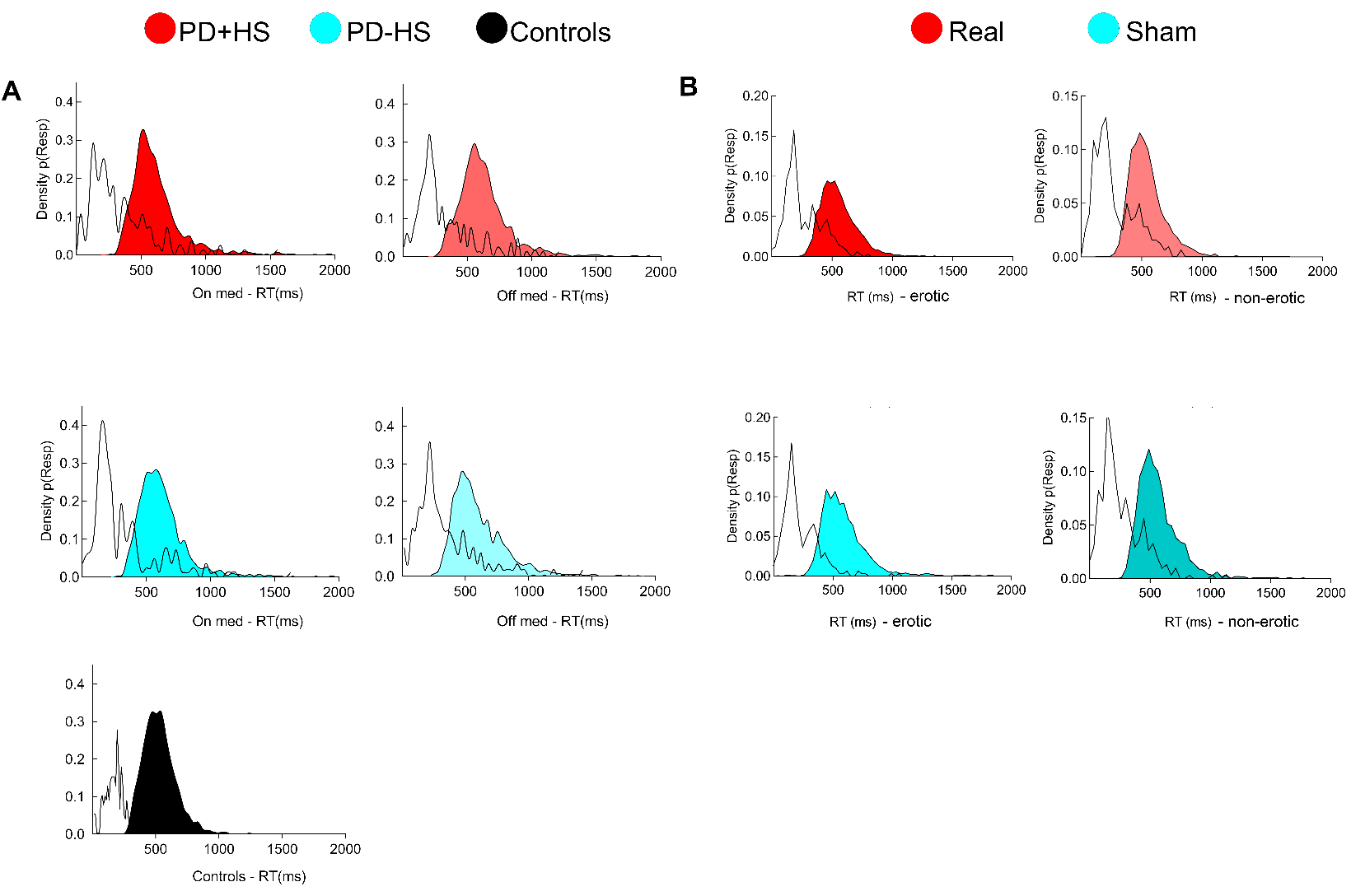
**Supplementary Fig. 4. Density distribution for StopRespond and Go conditions.**

**(A)** Density distribution for StopRespond (white distribution) and Go conditions (filled distribution) for PD+HS and PD-HS on and off medication as well as for healthy controls; **(B)** Density distribution for StopRespond (white distribution) and Go trials (filled distribution) per stimulation session (real or sham) and per trial type (erotic or non-erotic).

# Supplementary Tables

**Supplementary Table 1. Erotic stop signal reaction time results per *Medication* and *Group* conditions.**

|  | **PD+HS** | **PD+HS** | **PD-HS** | **PD-HS** | **Controls** |
| --- | --- | --- | --- | --- | --- |
|  | **ON** | **OFF** | **ON** | **OFF** |  |
| **Erotic** | | | | | |
| Go RT^a^ (ms) | 643.11±191.5 | 627.10±131.9 | 628.31±108.0 | 619.38±151.0 | 525.29±79.6 |
| StopRespond RT (ms) | 327.35±154.3 | 288.96±144.1 | 285.42±169.9 | 270.63±131.1 | 176.84±39.8 |
| p(stop)%^b^ | 64.03±17.4 | 66.11±9.2 | 66.72±16.6 | 69.30±17.0 | 69.54±13.8 |
| SSD^c^ (ms) | 232.92±108.6 | 277.65±117.2 | 251.93±121.2 | 243.59±133.7 | 251.57±97.1 |
| SSRT^d^ (ms) | 385.78±101.4 | 290.98±92.9 | 557.40±171.4 | 667.55±132.1 | 418.48±179.9 |
| ZRFT^e^ (ms) | 0.06±0.4 | 0.22±0.4 | -0.60±0.6 | -1.20±0.4 | -0.87±0.6 |
| Discrimination errors (total) | 2.07±1.2 | 2.86±3.3 | 2.73±4.1 | 2.93±3.6 | 2.44±5.8 |
| Premature errors | 0.64±1.6 | 0.43±0.9 | 0.20±0.5 | 0.0±0.0 | 0.13±0.3 |
| **Non-Erotic** |  |  |  |  |  |
| Go RT^a^ (ms) | 642.89±187.3 | 640.98±125.2 | 622.90±101.5 | 620.23±152.1 | 538.42±8.4 |
| StopRespond RT (ms) | 329.78±154.0 | 282.08±152.9 | 271.13±171.1 | 265.37±135.1 | 180.30±49.4 |
| p(stop)%^b^ | 65.21±15.1 | 67.85±14.0 | 69.36±17.9 | 69.86±17.4 | 69.05±15.7 |
| SSD^c^(ms) | 231.49±112.6 | 279.49±116.4 | 251.95±12.9 | 246.93±132.0 | 251.63±96.3 |
| SSRT^d^ (ms) | 381.78±122.0 | 380.79±72.6 | 559.37±134.1 | 677.71±152.6 | 445.42±159.9 |
| ZRFT^e^ (ms) | 0.08±0.5 | -0.08±0.3 | -0.70±0.6 | -1.12±0.4 | -0.94±1.7 |
| Discrimination errors (total) | 2.50±1.99 | 2.36±3.2 | 1.93±2.2 | 2.71±4.5 | 2.19±7.6 |
| Premature errors | 0.29±0.8 | 0.14±0.3 | 0.0±0.0 | 0.0±0.0 | 0.13±0.3 |

| ^a^*RT =* reaction time*;* ^b^*p(stop)% =* % probability of stopping*;* ^c^*SSD =* Stop Signal Delay*;* ^d^*SSRT:* Stop Signal Reaction Time*;* ^e^*ZRFT:* Z-score Relative Finish Time.  *PD+HS*: Parkinson’s Disease patients with hypersexuality; *PD-HS:* Parkinson’s Disease patients without hypersexuality. |
| --- |

**Supplementary Table 2. Stop signal reaction time results per group.**

|  | **PD+HS** | **PD-HS** | **Controls** | **Stats** |
| --- | --- | --- | --- | --- |
| Go RT^a^ (ms) | 509.07±81.1 | 513.38±79.4 | 459.26±81.4 | .130 |
| StopRespond RT (ms) | 249.98±146.7 | 198.27±68.2 | 161.70±35.1 | .045 |
| p(stop)%^b^ | 61.11±14.8 | 64.73±11.6 | 63.22±10.2 | .732 |
| SSD^c^(ms) | 236.67±125.9 | 260.27±102.1 | 239.97±102.4 | .826 |
| SSRT^d^ (ms) | 199.47±123.3 | 326.52±96.3 | 203.09±86.2 | .002 |
| Discrimination errors (total) | 6.13±5.4 | 5.57±6.9 | 2.94±2.4 | .201 |

| ^a^*RT =*reaction time*;*^b^*p(stop)% =*% probability of stopping*;*^c^*SSD =*Stop Signal Delay*;*^d^*SSRT:*Stop Signal Reaction Time. *PD+HS*: Parkinson’s Disease patients with hypersexuality;*PD-HS:* Parkinson’s Disease patients without hypersexuality. ^One-way ANOVA to test group effects on each variable. |
| --- |

**Supplementary Table 3. Successful vs unsuccessful inhibition under erotic condition.**

| **Tests and anatomical structures** | **X** | **Y** | **Z** | **T-value** |
| --- | --- | --- | --- | --- |
| **Medication** |  |  |  |  |
| *Superior parietal lobe* | -44 | -24 | 46 | 4.18** |
|  | -50 | -30 | 50 | 3.86** |
|  | -46 | -38 | 52 | 3.41** |
| *Anterior cingulate cortex (ACC)* | -12 | 10 | 40 | 3.75* |
| *Pre-supplementary motor area (pre-SMA)* | 6 | 20 | 56 | 3.41* |
| *Supplementary motor area* | 0 | -8 | 70 | 3.20* |
| **PD+HS on > off** |  |  |  |  |
| *Frontal pole* | 16 | 62 | 18 | 4.44** |
| *Anterior cingulate cortex (ACC)* | 0 | 24 | 14 | 4.13** |
| *Inferior-frontal gyrus* | 30 | 46 | 10 | 4.06** |
| *Supplementary motor area* | -4 | 6 | 44 | 3.69* |
|  | -6 | 6 | 56 | 3.34* |
|  | 10 | 6 | 50 | 3.27* |
| **PD+HS > PD-HS on** |  |  |  |  |
| *Superior parietal lobe* | -44 | -24 | 46 | 4.73** |
|  | -50 | -30 | 50 | 4.34** |
|  | -46 | -38 | 52 | 3.81** |
| *Pre-supplementary motor area (pre-SMA)* | -12 | 10 | 40 | 4.21** |
|  | 6 | 20 | 56 | 3.81** |
|  | -24 | 14 | 62 | 3.59* |

Tests and anatomical structures for the successful vs unsuccessful stop trials contrast in the erotic condition. *P<*.05 FWE cluster-wise corrected; **P<*.005; ***P<*.001. *PD+HS*: Parkinson’s Disease patients with hypersexuality*; PD-HS:* Parkinson’s Disease patients without hypersexuality.

**Supplementary Table 4.** **Successful inhibition vs. Go trials in the erotic condition.**

| **Tests and anatomical structures** | **X** | **Y** | **Z** | **T-value** |
| --- | --- | --- | --- | --- |
| ***Group*** |  |  |  |  |
| *Motor cortex* | 20 | -36 | 60 | 5.71** |
| *Cerebellum* | 24 | -16 | 52 | 5.18** |
|  | 20 | -44 | -28 | 4.52** |
|  | 16 | -34 | -32 | 3.66** |
| **PD-HS > PD+HS off** |  |  |  |  |
| *Cingulate* | 18 | -10 | 46 | 5.77 |
| *Superior parietal lobe* | 26 | -24 | 50 | 4.91 |
| *Motor cortex* | 20 | -36 | 62 | 4.5 |

Tests and anatomical structures for the successful (*stop-inhibit)* vs *go* trials contrast in the erotic condition. *P<*0.05 *FWE* cluster-wise corrected; **P<*.005; ***P<*.001.  *PD+HS*: Parkinson’s Disease patients with hypersexuality*; PD-HS:* Parkinson’s Disease patients without hypersexuality.

**Supplementary** **Table 5. Behavioral results of the stop signal task (study 2) after real and sham intermittent theta burst stimulation.**

|  | **Real**  **Erotic** | **Sham**  **Erotic** | ***P-*value** | **Real**  **Non-erotic** | **Sham**  **Non-erotic** | ***P-*value** |
| --- | --- | --- | --- | --- | --- | --- |
| Go RT^a^ (ms) | 564.82±106.38 | 593.96±115.52 | .06 | 552.00±93.11 | 581.68±105.52 | .05 |
| StopRespond RT^a^ (ms) | 213.05±90.92 | 242.50±106.40 | .11 | 222.71±109.09 | 221.95±106.88 | .96 |
| p(stop)% ^b^ | 71.37±14.56 | 70.50±19.11 | .72 | 71.17±13.78 | 71.20±17.70 | .99 |
| SSD^c^ (ms) | 304.99±128.79 | 303.31±137.56 | .89 | 297.72±118.86 | 307.27±136.37 | .47 |
| SSRT (ms) | 341.51±138.42 | 392.47±155.06 | .02 | 352.12±182.19 | 398.13±204.05 | .04 |
| ZRFT (ms) | -0.56±0.54 | -0.56±0.63 | .98 | -0.58±0.66 | -0.63±0.69 | .59 |
| Discrimination errors (total) | 2.89±3.53 | 2.50±3.24 | .62 | 3.33±4.46 | 2.83±3.93 | .61 |
| Comparisons of task measures between real and sham sessions for erotic and non-erotic trials (*P-va*lue: paired t-tests). ^a^*RT =* reaction time*;* ^b^*p(stop)%*: % probability of stopping*;* ^c^*SSD*: Stop Signal Delay*;* ^d^*SSRT:* Stop Signal Reaction Time*;* ^e^*ZRFT:* Z-score Relative Finish Time. | | | | | | |
